# A poly(A)-independent PacBio strategy for organellar transcriptome profiling

**DOI:** 10.1093/plphys/kiag596

**Published:** 2026-08-12

**Authors:** Xiaolin Gu, Jie Wang, Liyun Nie, Shuo Zhang, Yi Zou, Zhiqiang Wu

**Affiliations:** State Key Laboratory of Tropical Crop Breeding, Shenzhen Branch, Guangdong Laboratory of Lingnan Modern Agriculture, Key Laboratory of Synthetic Biology, Ministry of Agriculture and Rural Affairs, Agricultural Genomics Institute at Shenzhen, Chinese Academy of Agricultural Sciences, Shenzhen 518124, China; State Key Laboratory of Tropical Crop Breeding, Shenzhen Branch, Guangdong Laboratory of Lingnan Modern Agriculture, Key Laboratory of Synthetic Biology, Ministry of Agriculture and Rural Affairs, Agricultural Genomics Institute at Shenzhen, Chinese Academy of Agricultural Sciences, Shenzhen 518124, China; School of Medical, Molecular and Forensic Sciences, Murdoch University, Perth, WA 6155, Australia; State Key Laboratory of Tropical Crop Breeding, Shenzhen Branch, Guangdong Laboratory of Lingnan Modern Agriculture, Key Laboratory of Synthetic Biology, Ministry of Agriculture and Rural Affairs, Agricultural Genomics Institute at Shenzhen, Chinese Academy of Agricultural Sciences, Shenzhen 518124, China; School of Medical, Molecular and Forensic Sciences, Murdoch University, Perth, WA 6155, Australia; State Key Laboratory of Tropical Crop Breeding, Shenzhen Branch, Guangdong Laboratory of Lingnan Modern Agriculture, Key Laboratory of Synthetic Biology, Ministry of Agriculture and Rural Affairs, Agricultural Genomics Institute at Shenzhen, Chinese Academy of Agricultural Sciences, Shenzhen 518124, China; State Key Laboratory of Tropical Crop Breeding, Shenzhen Branch, Guangdong Laboratory of Lingnan Modern Agriculture, Key Laboratory of Synthetic Biology, Ministry of Agriculture and Rural Affairs, Agricultural Genomics Institute at Shenzhen, Chinese Academy of Agricultural Sciences, Shenzhen 518124, China; State Key Laboratory of Tropical Crop Breeding, Shenzhen Branch, Guangdong Laboratory of Lingnan Modern Agriculture, Key Laboratory of Synthetic Biology, Ministry of Agriculture and Rural Affairs, Agricultural Genomics Institute at Shenzhen, Chinese Academy of Agricultural Sciences, Shenzhen 518124, China

Dear Editor,

Understanding gene expression is fundamental to elucidating cellular functions and regulatory mechanisms. Although the nuclear genome has been extensively characterized, mitochondrial and chloroplast transcriptomes (organellar transcriptomes) remain comparatively underexplored despite their essential roles in energy metabolism, photosynthesis, and stress responses (Leister et al. 2017). Unlike nuclear transcripts, organelle RNAs often retain polycistronic transcription as a prokaryotic-like feature, while also undergoing extensive posttranscriptional processing and pervasive genome-wide transcription that reflect both bacterial ancestry and organelle-specific elaborations of gene expression (Zhelyazkova et al. 2012; Small et al. 2013; Castandet et al. 2019; Sanita Lima et al. 2024). Long-read RNA sequencing (RNA-seq) enables the direct acquisition of full-length transcripts, providing unprecedented opportunities to resolve complex organellar transcript structures (Koster et al. 2024). However, comprehensive characterization of organellar transcriptomes remains constrained by current library preparation strategies. Traditional RNA-seq workflows rely on poly(A) enrichment of mRNAs, yet poly(A) tails in plant organelles are primarily associated with RNA degradation rather than mature transcripts (Forsythe et al. 2022), resulting in inefficient capture of mature organellar RNAs. Therefore, optimizing library preparation strategies within the long-read RNA-seq to achieve efficient and comprehensive capture of organellar transcripts remains a critical challenge.

In this study, we optimized a long-read RNA-seq workflow, termed Poly(A)-independent sequencing (PAI-seq) (Fig. 1a). In PAI-seq, highly abundant rRNAs are first removed from total RNA, followed by ligation of a defined 3′ adaptor, adapted from the fixed-sequence strategy used in small RNA library construction (Lu et al. 2007). An adaptor-specific primer is then used to initiate reverse transcription, replacing the conventional oligo(dT)-primed strategy and enabling cDNA synthesis independently of poly(A) tails without introducing significant 3′ end bias (Fig. 1b). This design preserves both polyadenylated and non-polyadenylated RNAs, thereby preventing the systematic loss of organellar transcripts and enabling simultaneous profiling of nuclear and organellar transcriptomes from the same RNA sample. Combined with the Kinnex library preparation workflow, PAI-seq consistently generated high-quality long-read transcriptome data. The *Arabidopsis thaliana* Col-0 dataset comprised 3.19 to 5.58 million reads with mean read lengths of 1,512.2 ± 831.0 to 1,820.7 ± 689.4 bp, whereas the *Oryza sativa* cv. Nipponbare dataset comprised 6.23 to 14.65 million reads with mean read lengths of 1,566.5 ± 554.2 to 1,898.5 ± 745.8 bp, providing sufficient read lengths, with most reads spanning nearly the entire annotated coding sequence (CDS) (median CDS completeness of 0.99 to 1.00; Fig. S1), thereby enabling comprehensive characterization of organellar transcript structures. To assess its performance, we systematically compared 3 long-read RNA-seq library preparation strategies in *A. thaliana* and *O. sativa*: (i) traditional poly(A) tail RNA-seq (poly(A)-based long-read RNA-seq), (ii) poly(A) tail RNA-seq with rRNA depletion (ribo-depleted long-read RNA-seq), and (iii) PAI-seq. Compared with the other 2 methods, PAI-seq provided a more comprehensive view of organellar RNA populations and broader opportunities for downstream analyses.

**Figure 1:**
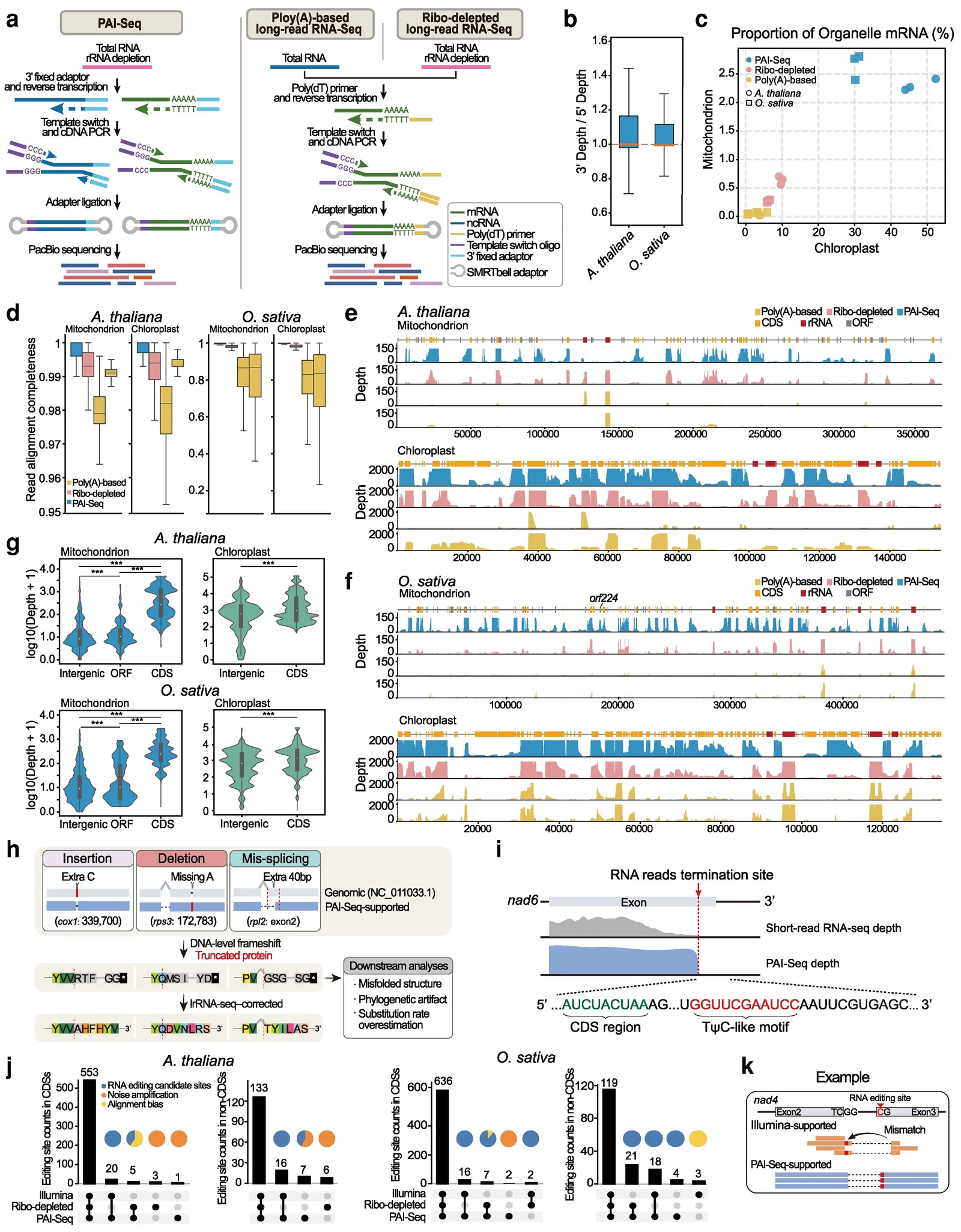
Comparative analysis of RNA library preparation strategies. a) Schematic of the optimized long-read RNA-seq workflow. b) Assessment of 3′ and 5′ coverage bias in organellar transcripts generated by PAI-seq (the ratio was calculated as the mean sequencing depth across the terminal 10% of the transcript at the 3′ end divided by that at the 5′ end. For each analysis, 5,000 primary reads were randomly sampled, and the analysis was repeated independently 100 times. The median value from the 100 replicates was used for each transcript). c) Proportion of non-rRNA organellar transcripts captured in *A. thaliana* and *O. sativa* under different library preparation strategies. d) Comparison of mapping rates of organellar reads across different strategies (read completeness calculated as the alignment length divided by the total read length). e) Transcript coverage depth profiles of *A. thaliana* organellar transcriptomes generated using different RNA library preparation strategies (depth normalized per million reads; average sequencing depth across 3 biological replicates; *y* axis thresholds: 2,000 for the chloroplast genome and 150 for the mitochondrial genome). f) Transcript coverage depth profiles of *O. sativa* organellar transcriptomes generated using different RNA library preparation strategies (depth normalized per million reads; average sequencing depth across 3 biological replicates; *y* axis thresholds: 2,000 for the chloroplast genome and 150 for the mitochondrial genome). g) Violin plots showing transcript coverage depth across different genomic regions in *A. thaliana* and *O. sativa* organelles under the PAI-seq workflow (depth normalized per million reads). h) Refinement of organellar genome annotations facilitated by PAI-seq. i) Advantages of PAI-seq in resolving transcription termination sites. j) Comparison of candidate RNA editing sites detected under different RNA library preparation strategies (pie charts summarize the major causes of discordant RNA editing site detection among strategies). k) PAI-seq improves transcript boundary resolution compared to short-read RNA-seq.

PAI-seq substantially improved organellar transcript capture. To avoid overestimating organellar transcript abundance due to residual organellar rRNA, we quantified organellar transcripts as the mean proportion of non-rRNA mitochondrial or chloroplast reads relative to total library reads for each library type. Compared with traditional poly(A)-based long-read RNA-seq, it increased mitochondrial transcript capture by ∼74-fold and chloroplast transcript capture by ∼14.3-fold in *A. thaliana*, and by ∼21.5-fold and ∼6-fold, respectively, in *O. sativa* (Fig. 1c). By comparison, ribo-depleted long-read RNA-seq increased mitochondrial transcript capture by ∼20.5-fold and chloroplast transcript capture by ∼2.9-fold in *A. thaliana*, and by ∼5.5-fold and ∼2.2-fold, respectively, in *O. sativa*. Moreover, we selected 2 publicly available poly(A)-based long-read RNA-seq datasets for *A. thaliana* and *O. sativa* for subsequent comparative analyses. Across all datasets, PAI-seq consistently achieved the highest organellar read mapping rates, with individual organellar reads showing alignment ratios >99% in both *A. thaliana* and *O. sativa*, outperforming the other 2 sequencing strategies (Fig. 1d).

PAI-seq markedly improved organellar genome coverage by transcriptomic reads. Under poly(A)-based long-read RNA-seq, mitochondrial genome coverage remained only ∼4% to 8% in *A. thaliana* and ∼22% to 27% in *O. sativa* (defined as ≥10 reads). Ribo-depleted long-read RNA-seq increased mitochondrial genome coverage to ∼63% in *A. thaliana* and ∼55% in *O. sativa*, whereas PAI-seq further increased mitochondrial genome coverage to 82% and 92%, respectively. Notably, although all libraries were generated from the same probe-based rRNA-depleted RNA, organellar rRNA remained abundant in poly(A)-based long-read RNA-seq (Fig. 1e and f). Residual rRNA after probe-based depletion is expected (Castandet et al. 2016), whereas its enrichment in poly(A)-based libraries likely reflects oligo(dT) capture of polyadenylated rRNA decay intermediates (Holec et al. 2006). Plant mitochondrial genomes contain extensive noncoding regions (Wang et al. 2024), many of which exhibit pervasive transcription (Sanita Lima et al. 2024). However, transcript abundance in these regions was generally much lower than that of coding sequences (Fig. 1g). Similarly, most annotated mitochondrial ORFs exhibited low transcript coverage, although a subset showed relatively high transcriptional activity, indicating that they may warrant further investigation (Table S1). For example, *O. sativa* mitochondrial *orf224* has been implicated in cytoplasmic male sterility (Fujii et al. 2010).

One of the major advantages of long-read RNA-seq is its ability to accurately resolve transcript structures (Monzó et al. 2025), provided that sufficient transcript completeness and sequencing depth are achieved, which are effectively supported by PAI-seq. The high-quality full-length transcripts allowed precise identification and correction of insertion/deletion and splice junction annotation errors, which would otherwise systematically compromise CDS annotation accuracy and downstream functional and evolutionary analyses (Fig. 1h). In addition, PAI-seq enabled a more comprehensive characterization of organellar transcripts, which are governed by complex transcriptional and posttranscriptional regulatory mechanisms. For instance, PAI-seq identified a reproducible alternative 3′ transcript end in the mitochondrial gene *nad6*, located upstream of the annotated stop codon (Fig. 1i). Compared with short-read RNA-seq, long-read RNA-seq precisely resolved the transcription termination site, marked by a sharp decrease in read coverage. Further sequence analysis identified a TψC-like motif downstream of this 3′ end, a hallmark of mitochondrial t-elements. This feature suggests that the observed transcript terminus may be generated through t-element-directed posttranscriptional processing (Forner et al. 2007; Yu et al. 2025).

In organelles, long-read RNA-seq enables characterization of RNA editing by simultaneously capturing multiple co-occurring editing sites within individual transcripts (Liu et al. 2023). However, its application has been limited by insufficient coverage of organellar RNAs. By substantially improving organellar transcript coverage, PAI-seq substantially mitigates this limitation and enables more comprehensive analyses of organelle RNA editing. To evaluate this potential, we compared long-read RNA-seq (PAI-seq and ribo-depleted long-read RNA-seq) with short-read RNA-seq for the identification of candidate RNA editing sites. For high-frequency editing sites, the 3 approaches showed concordance. In contrast, detection of low-frequency sites (editing efficiency < 20%) was highly sensitive to sequencing depth and threshold settings (Fig. 1j). Moreover, candidate RNA editing sites located near splice junctions may be missed in short-read data due to the limited capacity of short reads to reliably span splice boundaries and the inherent limitations of alignment algorithms (Steijger et al. 2013); this limitation is effectively resolved by long-read RNA-seq, as exemplified by *nad4* (NC_037304.1: 210033) and *nad7* (NC_037304.1: 242625) in the *A. thaliana* mitochondrial genome, and *ycf3* (NC_001320.1: 42013) in the *O. sativa* chloroplast genome (Fig. 1k). However, even with PacBio HiFi long-read RNA-seq, reliable identification of low-frequency RNA editing events depended on sufficient sequencing depth and stringent variant-calling thresholds. This limitation was evident in the ribo-depleted long-read RNA-seq libraries generated in this study, where low organellar read coverage reduced the sensitivity for detecting RNA editing sites (Fig. 1j).

In summary, PAI-seq substantially improves organellar RNA capture, mapping quality, and organellar genome coverage, enabling comprehensive analyses of the transcriptional landscapes of both coding and noncoding regions. It provides an efficient and broadly applicable strategy for long-read transcriptome profiling.

## Supplementary Material

kiag596_Supplementary_Data

## Data Availability

The raw sequence data reported in this paper have been deposited in the Genome Sequence Archive in the National Genomics Data Center (GSA: CRA043544) that are publicly accessible at https://ngdc.cncb.ac.cn/gsa. The poly(A)-based long-read RNA-seq datasets used for the initial comparison of organellar read capture efficiency were downloaded from the NCBI Sequence Read Archive, and the corresponding references are provided in Table S2. For *A. thaliana*, the accession numbers were SRR22719002, SRR22719003, SRR22719004, SRR22719005, SRR22719006, SRR22719007, and SRR14584394. For *O. sativa*, the accession numbers were CRR836528, SRR12057535, SRR18294823, SRR18961263, and SRR15851185. Based on average read length, sequencing yield, and the number of organellar reads, representative datasets were selected for subsequent comparative analyses, including SRR14584394 and SRR22719003 for *A. thaliana*, and SRR12057535 and SRR15851185 for *O. sativa*.
