# Supplementary material for: A poly(A)-independent PacBio strategy for organellar transcriptome profiling
Source: Plant Physiol. 2026 Aug 12;201(4):kiag596. doi: 10.1093/plphys/kiag596 (PMC13521279; doi:10.1093/plphys/kiag596)
Supplement: Supplementary file 1 [file kiag596_supplementary_data.zip › Fig. S1.docx]

**Fig. S1** Per-read CDS completeness across library types in organellar transcriptomes (CDS completeness was calculated as the ratio of annotated CDS covered by each read; a value of 1.0 indicates complete coverage of the annotated CDS).
